# Fracture Response of X80 Pipe Girth Welds under Combined Internal Pressure and Bending Moment

**DOI:** 10.3390/ma16093588

**Published:** 2023-05-07

**Authors:** Li Zhu, Naixian Li, Bin Jia, Yu Zhang

**Affiliations:** 1 School of Civil Engineering and Architecture, Southwest University of Science and Technology, Mianyang 621010, China; zhuli@swust.edu.cn (L.Z.);; 2 Sichuan Deyuan Pipeline Technology Co., Ltd., Chengdu 610041, China

**Keywords:** fracture, internal pressure, bending moment, girth welds, pipeline

## Abstract

In order to determine the effect of defect size on the pipeline fracture performance of girth welds in oil and gas pipelines, ABAQUS was used to simulate the fracture responses of X80 pipelines with girth weld defects under internal pressure and bending moment conditions based on damage mechanics. In particular, the length and depth of defects were parametrically studied; the defect depth range was 20–80% of the wall thickness, and the circumferential length range of the defects was 5–20% of the pipeline circumference. The results show that, under the combined action of internal pressure and bending moment, the defect depth was more associated with adverse effects than the circumferential length of the defect. The failure load did not linearly decrease as the size of the defect increased, but when the depth of the defect reached a certain value, the failure load suddenly decreased.

## 1. Introduction

Due to their high capacity, low cost, ease of use, and efficiency, high-grade steel pipelines are utilized extensively for the transportation of oil and gas. Steel pipes are typically manually welded together every 12 m for long-distance pipeline transmission. Due to the impact of factors, such as construction environment, it is inevitable that defects will occur in the girth welds; welds are often the point of structural fracture [1,2]. Moreover, the environments in which pipeline laying occur are extremely complex, which creates great safety challenges in association with girth welds. For example, a single pipeline may cross many geological environments, such as valleys, rivers, deserts, and permafrost. Internal pressure and bending moment are the main load forms for pipelines and may cause fracture of the pipeline girth weld defects. Although there are some relevant standards for evaluating pipeline fractures, some of them were mainly established for the elastic stage and are not applicable to the ductile fracture of pipeline girth welds [3,4], while the others are based on a semi-empirical and a semi-theoretical formula, which is used to evaluate the internal pressure explosion of pipelines with external corrosion defects [5,6]. However, most girth weld defects are internal, and their fractures occur in the plastic stage. As a result, the above-mentioned codes and standards are not applicable. Therefore, a fracture evaluation method suitable for pipeline deformation capacity is required.

Østby et al. [7] proposed a strain-based design method, which can be used to determine the allowable strain without considering material properties and crack geometry. However, it is difficult to capture the local plastic deformation and fracture of pipelines [8]. Research on pipe fracturing is mainly based on traditional fracture mechanics and damage mechanics; the J-integral and CTOD (crack-tip opening displacement) are two very important fracture parameters in fracture mechanics. These two parameters characterize the severity of unstable crack propagation at the crack tip and can be used to evaluate a crack’s growth state [9]. However, if a crack has destabilized, the propagated J-integral theory is no longer applicable [10]. In order to solve this problem, a J-integral R-curve method for unstable cracks has been proposed [11,12]. The theory of fracture mechanics has been widely used in the study of pipeline failure. For example, the collapse behavior of a thick-walled pipe was studied considering the combined effect of tension and the bending moment [13]. Crack assessment for girth-welded pipes with surface and embedded cracks was provided using a strain-based CTOD method [14,15]. A three-dimensional, nonlinear elastic–plastic model was established to study the effects of girth welds on offshore pipelines [14,15,16,17,18]. These studies were all based on traditional fracture mechanics. However, fracture mechanics, which must be studied based on initial cracking, impose significant limitations on the study of pipe failure. Damage mechanics models can effectively address the limitations of fracture mechanics and have been widely used for metal fracture [16,17,18,19]. As pipelines are metal structures, damage mechanics theory is also very applicable to pipeline engineering and has been studied by many scholars. Oh et al. [20,21,22,23] formulated a series of notch tensile specimens for X65 pipeline steel and developed a GTN (Gurson–Tvergaard–Needleman) model and an uncoupled fracture model, which were used to analyze the results of a full-size pipeline blast test. Saneian and Han [24,25,26] established an MMC (Mohr–Coulomb) model of an X80 pipeline and used this model to analyze the fracture response of a pipeline with corrosion defects under complex loads. 

Specifically, for girth welds, the GTN damage model was used to evaluate the failures associated with different strength-matching and size parameters of welds under tensile load [27]. However, the damage parameters were all determined from the base metal; thus, the difference in the damage parameters between the weld and the base metal was not considered, and nor was the difference caused by different strength-matching parameters. Based on the net section collapse criterion, Jin et al. deduced the limit load expression of girth welds for defects with different size parameters under various load combinations and verified it using the finite element method [28]. Lu et al. [29] obtained the equation of the residual strength of an incomplete full penetration defect model at the root of a girth weld pipe via the stress function method, which was in good agreement with the finite element results. According to the flow stress failure criterion, a stress analysis was performed on a pipe girth weld under extreme compression [30]. In general, the current research on the failure of pipe girth welds focuses mainly on the specification design and fracture mechanics. Because traditional specification design is too conservative to fully explore the performance of the material, and fracture mechanics studies must be based on cracking, the scope of research has been greatly limited; the use of damage mechanics offers a good solution to the shortcomings of the traditional method, and the application of damage mechanics to girth weld research is still lacking. 

In this paper, an uncoupled fracture model of an X80 pipeline girth weld was established to analyze the fracture responses of girth weld defects of different sizes under internal pressure and bending moment conditions. The uncoupled fracture criterion is the failure of a material when it reaches the critical strain of the stress state to which it is subjected. Using this model, a parameterized study was conducted on the influence of defect size on pipeline failure. The research results can provide a guide for the risk assessment of girth weld defects under different internal pressure and bending moment conditions.

## 2. Ductile Fracture Model

Previous studies [31,32,33,34] have shown that stress triaxiality and the Lode parameters are the two main parameters that affect fracture strain, and these have been extensively applied to characterize the stress states of materials. Stress triaxiality with Lode parameters is expressed as follows:

(1) $$\eta =\frac{\sigma_{m}}{\bar{\sigma }}$$

(2) $$\bar{\theta }=1-\frac{2}{\pi }\arccos\frac{27J_{3}}{2{\bar{\sigma }}^{3}}$$

where η, $\bar{\theta }$, $\sigma_{m}$, $\bar{\sigma }$, and $J_{3}$ are the stress triaxiality, dimensionless Lode angle parameter, mean stress, equivalent stress, and third invariant of deviatoric stress, respectively.

On the basis of the stress-modified critical strain model (SMCS) that only takes into account stress triaxiality in fracture strain, Huang et al. [35] developed the LSMCS model (stress-modified critical strain model considering the effect of Lode parameters), considering the influence term of the Lode parameters as follows:

(3) $${\bar{\varepsilon }}_{f}^{\mathrm{pl}}=\alpha \exp(-1.5\eta )[\gamma +(1-\gamma ){\bar{\theta }}^{2}]$$

where *α* and *γ* are the two material parameters to be determined, and ${\bar{\varepsilon }}_{f}^{\mathrm{pl}}$ is the fracture strain.

## 3. Calibration Procedure

### 3.1. Experimental Method

To obtain the fracture parameters of the X80 pipeline girth welds, we first needed to determine the constitutive model of X80 pipeline steel. For X80 pipeline steel, standard tensile specimens (SRB) were designed according to the requirements of GB/T+228.1-2010 [36].

**Figure 1:**
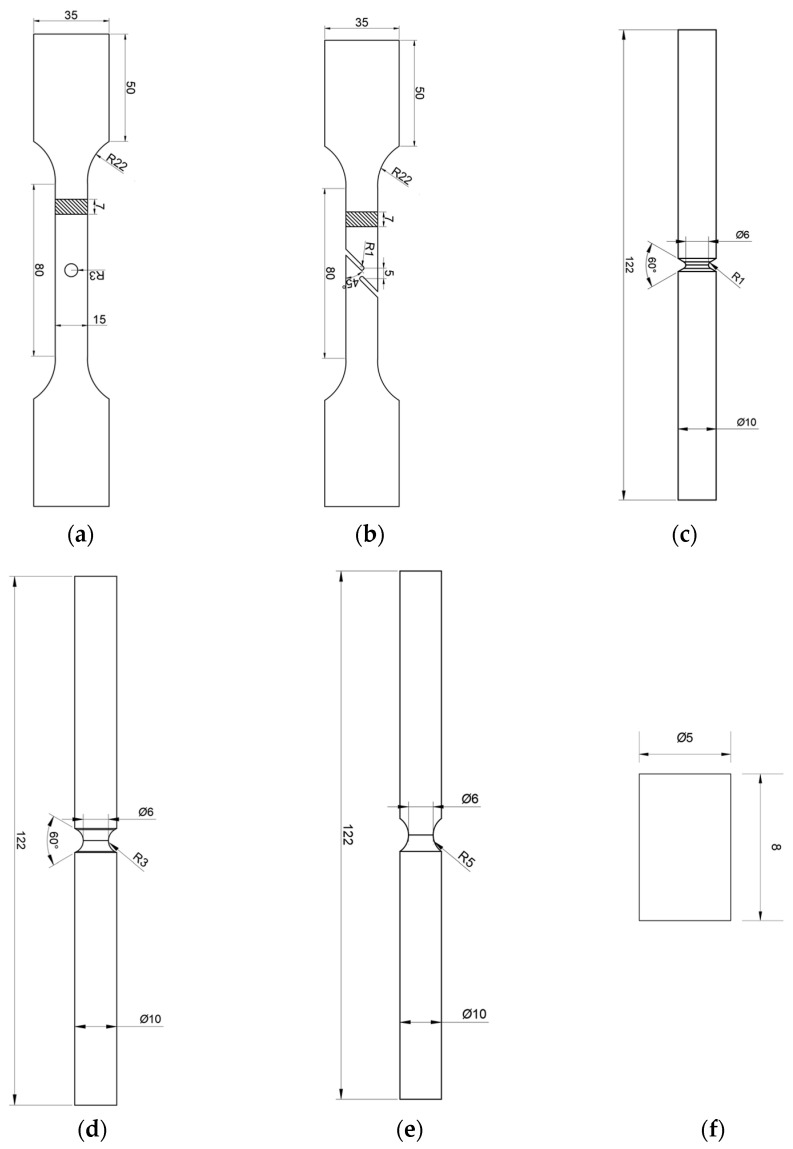
Specially shaped weld specimens (mm): (**a**) CH6 (central hole tensile specimen, round hole diameter: 6 mm), (**b**) FS (flat shear specimen), (**c**) NRB1 (notched round bar tension specimen, radius: 1 mm), (**d**) NRB3 (notched round bar tension specimen, radius: 3 mm), (**e**) NRB5 (notched round bar tension specimen, radius: 5 mm), (**f**) compression.

**Figure 2:**
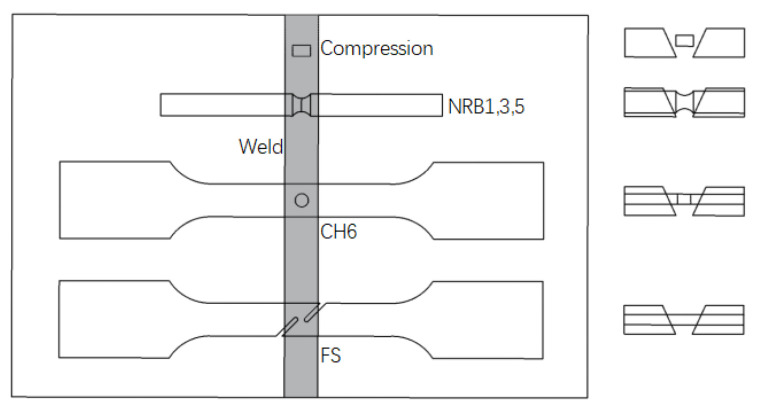
Schematic diagram of weld specimen pick-up.

The welding material (91T8) in this work was taken from an X80 girth-welded pipe with a diameter of 1219 mm and a wall thickness of 18.4 mm. The welding method used was in accordance with GB/T 31032-2014 [37]. The fluxed cored arc welding (FCAW) method was used for welding. The welding parameters were as follows: (1) welding current of 170 Amps, (2) welding voltage of 20 V, (3) wire feed speed of 5 m/min, and (4) front arc energy of 0.72 kJ/mm. In order to ensure that the fracture position would occur at the weld, five different specially shaped test pieces were designed as shown in Figure 1. All specimens were taken from the longitudinal direction of the pipe. The girth weld was located at the notch of the specially shaped specimens, as shown in Figure 2.

The experiment was performed on a CMT5150 universal test machine with a maximum load of 100 kN. The distance of the extensometer was 50 mm with an accuracy grade of 1. All experiments were performed at room temperature with a loading rate of 0.45 mm/min. 

### 3.2. Constitutive Model

#### 3.2.1. X80 Pipeline Steel

**Figure 3:**
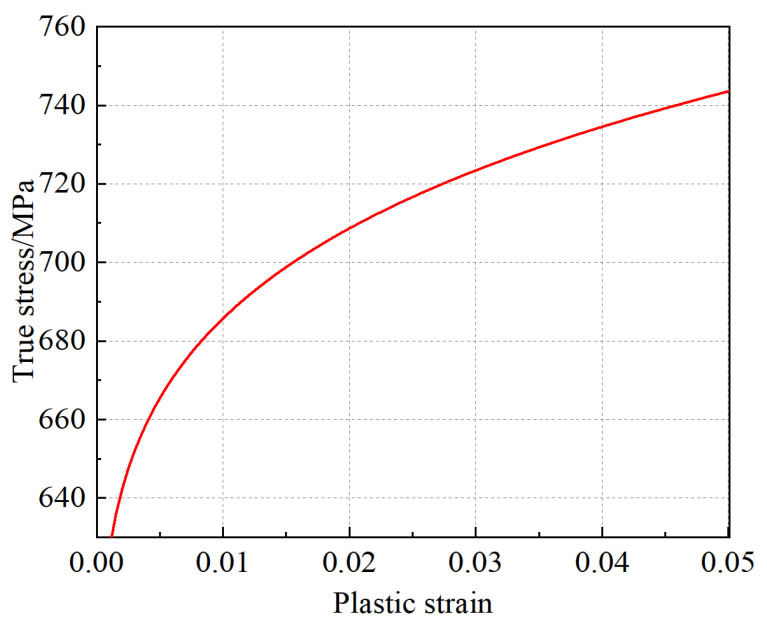
Johnson–Cook hardening curve.

The mechanical property parameters determined for the X80 pipeline steel are shown in Table 1. The yield strength was determined by a 0.2% plastic offset (Rp0.2). The hardening curve was described by a static Johnson–Cook model [38]:

(4) $$\bar{\sigma }=A+B{\left({\bar{\varepsilon }}^{\mathrm{pl}}\right)}^{n}$$

where ${\bar{\varepsilon }}_{}^{\mathrm{pl}}$ is the equivalent plastic strain; *A*, *B*, and *n* were determined experimentally as 506.94 MPa, 398.467 MPa, and 0.17402, respectively. The hardening curve is shown in Figure 3.

#### 3.2.2. X80 Pipeline Weld

**Figure 4:**
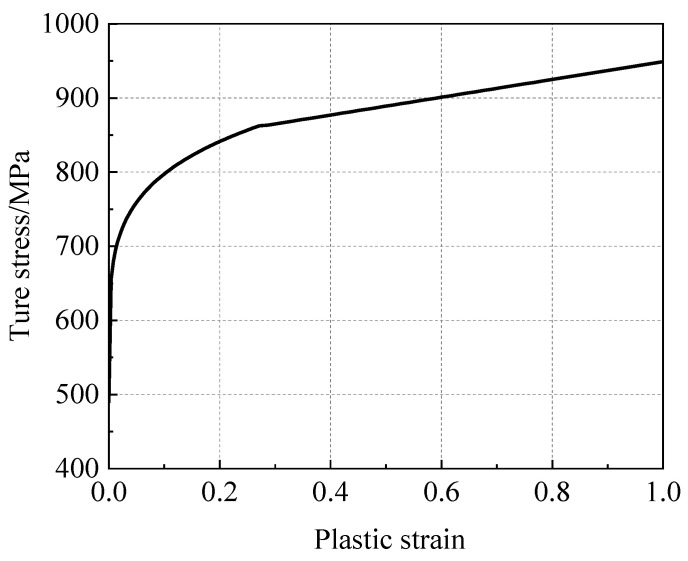
X80 girth weld stress–strain curve.

According to previous studies [39,40,41,42], the mechanical properties of the elastic stage of a girth weld are consistent with those of the base metal, which was also considered in this work. Therefore, in this work, it was necessary to determine the hardening curve of the girth weld of an X80 pipeline. The hardening constitutive relationship of the girth weld was determined using compression specimens. The obtained hardening curve is shown in Figure 4. The curve is described by the following subsection function:

(5) $$\left\{ \begin{array}{l} \bar{\sigma }=490.2+478.1{\left({\bar{\varepsilon }}^{\mathrm{pl}}\right)}^{0.1918}(0<{\bar{\varepsilon }}_{}^{\mathrm{pl}}<0.272)\\ \bar{\sigma }=120{\bar{\varepsilon }}_{}^{\mathrm{pl}}+829(0.272<{\bar{\varepsilon }}_{}^{\mathrm{pl}}) \end{array}\right.$$

### 3.3. Damage Model

#### 3.3.1. Identification of Fracture Parameters

**Figure 5:**
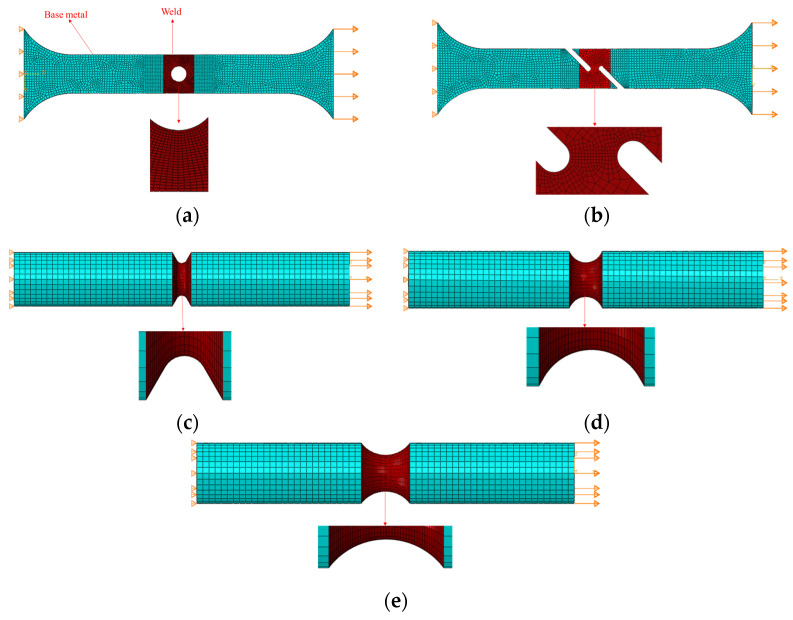
Finite element mesh model: (**a**) CH6, (**b**) FS, (**c**) NRB1, (**d**) NRB3, (**e**) NRB5.

According to the elastic–plastic finite element model, the fracture strain, stress triaxiality, and Lode parameters of different types of X80 girth welds were analyzed and calibrated. Three-dimensional finite element models of the specially shaped notch tensile specimens were established. During the experimental process, large deformation occurred at the weld gap. The heat-affected zone was still in the elastic stage, or that in the plastic stage was small enough to be neglected. Thus, the material softening of the heat-affected zone was ignored, considering the properties of the base material. Notch position was endowed with the attributes of the weld material. The weld was considered as an isotropic and a uniform material [43]. One end of the specimen was articulated, while the other end was loaded with displacement, as shown in Figure 5. A C3D8R element was used, and the gap part was encrypted. The calculations were carried out using ABAQUS/ Explicit with reduced integral control.

#### 3.3.2. Parametric Identification for the LSMCS Model

**Figure 6:**
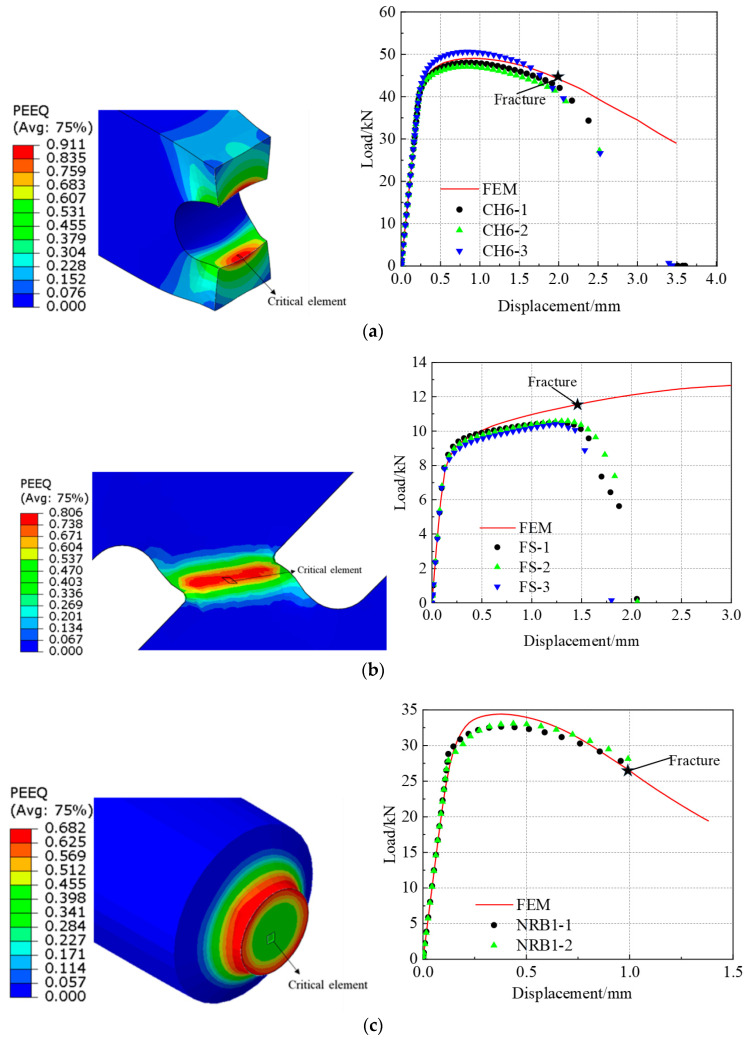

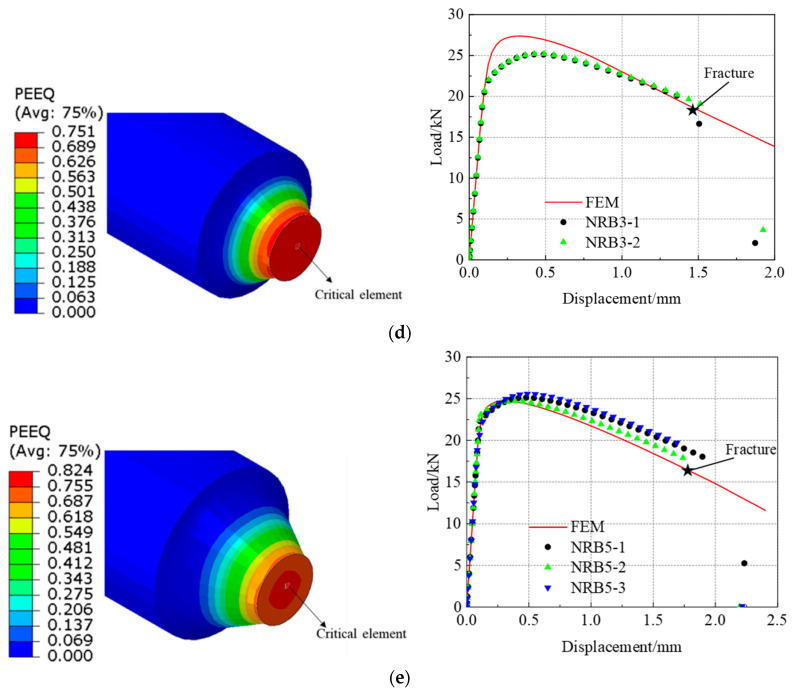
Determination of critical displacement: (**a**) CH6, (**b**) FS, (**c**) NRB1, (**d**) NRB3, (**e**) NRB5.

The load displacement curves that were drawn based on the finite element and experimental results are shown in Figure 6. The established base material and the girth weld constitutive models were able to reflect the mechanical behaviors of the weld tensile specimens under different stress states. The initial fracture points of the specimens are marked with stars in Figure 6. The experimental results revealed that the notched round bars cracked in the centers of the specimens, while the plate-patterned specimens cracked on the notched surface. The equivalent plastic strain of the critical element under the corresponding fracture displacement of the test was the fracture strain. 

**Figure 7:**
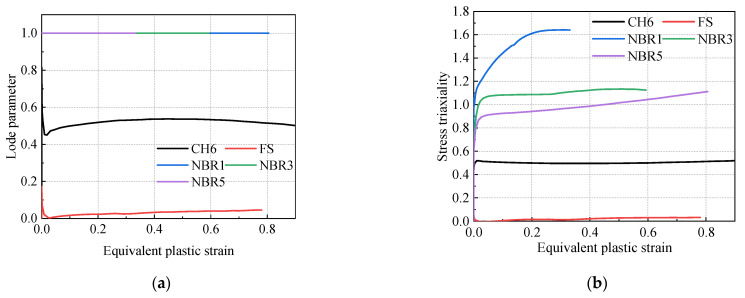
Evolution of equivalent plastic strain and stress states: (**a**) evolution of equivalent plastic strain and Lode parameters; (**b**) evolution of equivalent plastic strain and stress triaxiality.

The element body at the fracture position was selected to evaluate the evolution processes of stress triaxiality, Lode parameters, and equivalent plastic strain, as shown in Figure 7. It was observed that the Lode parameter of the round bar specimen remained at 1. It was found that the Lode parameter of the round hole plate in the process of tensile change was near 0.5, while that of the shear specimen remained near 0. Furthermore, the stress triaxiality of the sheet metal specimens remained essentially unchanged during the tensile process, while the notched round bar tension specimen and tensile specimens all increased at different levels. The stress triaxiality of specimens with radii of 1 mm was greatly increased. The stress states of the samples changed constantly during the whole tensile process, so the triaxial stress triaxiality of the element had to be averaged with the Lode parameters as follows [32,34]:

(6) $${\left(\eta \right)}_{\text{av}}=\frac{1}{{\bar{\varepsilon }}_{\text{f}}^{\text{pl}}}\int_{0}^{{\bar{\varepsilon }}_{\text{f}}^{\text{pl}}}\eta d{\bar{\varepsilon }}^{\text{pl}}$$

(7) $$(\bar{\theta }{)}_{\mathrm{av}}=\frac{1}{{\bar{\varepsilon }}_{f}^{\mathrm{pl}}}\int_{0}^{{\bar{\varepsilon }}_{f}^{\mathrm{pl}}}\bar{\theta }d{\bar{\varepsilon }}^{\mathrm{pl}}$$

where ${\left(\eta \right)}_{\mathrm{av}}$ is the average stress triaxiality, $(\bar{\theta }{)}_{\mathrm{av}}$ is the average Lode parameter, and ${\bar{\varepsilon }}_{f}^{\mathrm{pl}}$ is the fracture strain.

**Figure 8:**
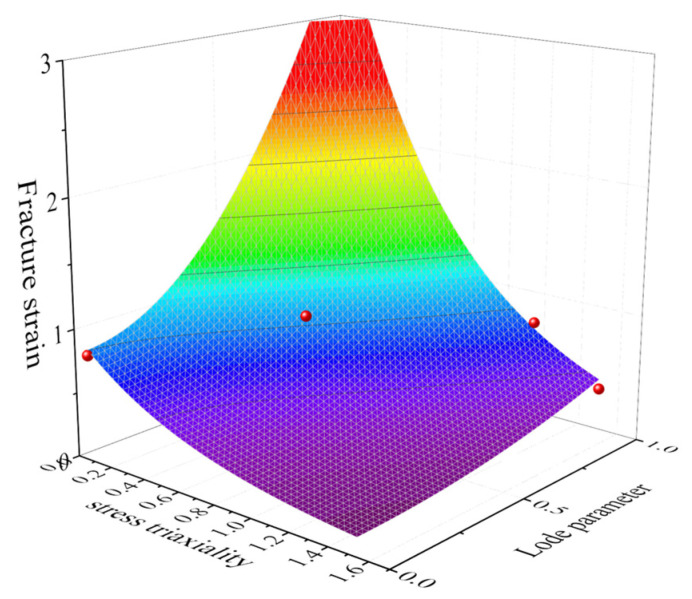
Calibration fracture surface for the LSMCS criterion.

Based on the results obtained using a FEM (finite element model) and Equations (6) and (7), the LSMCS model was fitted by determining the fracture parameters with α as 3.883 and γ as 0.218. The model fracture surface is shown in Figure 8.

## 4. Accuracy Verification

**Figure 9:**
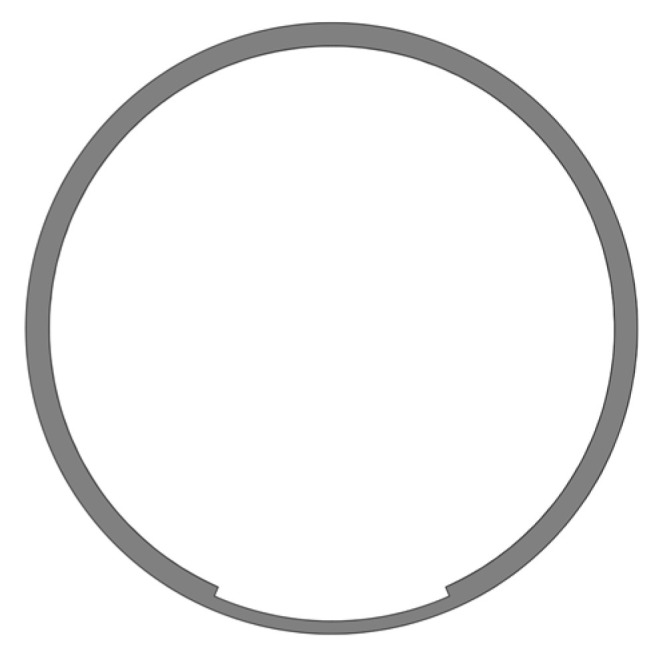
Schematic diagram of test pipe defects.

First, to validate the effectiveness and precision of the established fracture model in predicting pipeline fracture, a full-scale blast test of the reference pipeline [44] was selected, and a corresponding numerical model was established to compare the blast pressure. The diameter, thickness, and length of the test pipe were 1219 mm, 18.4 mm, and 12,000 mm, respectively. The girth weld defect was located on the inner surface of the pipe, and the circumferential length (L), width (W), and depth (D) of the defect were 500 mm, 1 mm, and 9.2 mm, respectively, as shown in Figure 9. The girth weld was located in the middle of the pipe. The width of the pipe weld was set at 15 mm, which was much wider than the defect width. Moreover, the weld width had no effect on the pressure at which the pipe burst.

**Figure 10:**
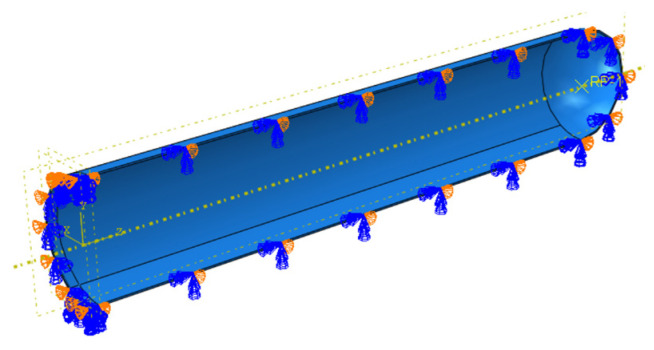
Application of symmetric boundary conditions.

A finite element model corresponding to the test pipe was established in ABAQUS. The pipe model with three-dimensional solid elements was the best suited for this method [45]. The quarter model was used to save computing time by applying symmetric constraints on the corresponding symmetric plane, as shown in Figure 10. 

**Figure 11:**
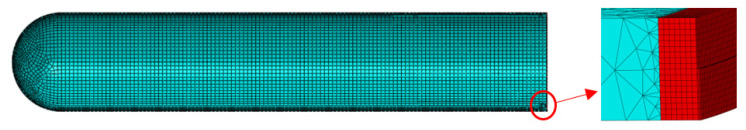
The pipe showing the details of the crack.

The element partition in the defect was much more intensive than that of the pipe as a whole. The element size in the encrypted part was 1 mm, and the element length in the defect was 0.1 mm, with a total of 95,678 elements. In Figure 11, the red area is the girth weld, while the blue area is the base metal. Due to the existence of defects, the girth weld inevitably had a stress concentration at the defects [30], leading to fracture. A large deformation occurred mainly in the defects, thus allowing the softening effect of the heat-affected zone to be ignored [28,29]. Thus, the model was composed of base metal and weld metal. The material attributes of the pipe base metal and weld were assigned, respectively. The material attributes of the weld included the LSMCS fracture model, which was defined in ABAQUS by modifying the keywords in the INPUT file [45], which characterized the pipeline fracture by removing the failure element. Pressure was applied to the inner wall of the pipe until the weld defect fracture occurred. Quasi-static calculations were performed using the ABAQUS/Explicit dynamic, reduced integral control, and the C3D8R element.

**Figure 12:**
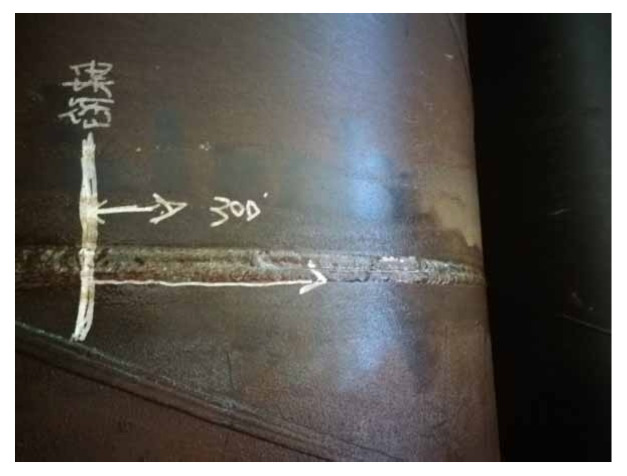
Failure modes of the test pipe [44].

Figure 12 shows the failure pattern of test pipe after blasting. It can be seen that the weld defect penetrated the girth of the pipe along the crack and formed a fractured girth trace. When the internal pressure of the pipe was pressurized to 21.27 MPa, the pipe leaked at the prefabricated defect of the girth weld, and the maximum pressure during blasting was 21.36 MPa. 

**Figure 13:**
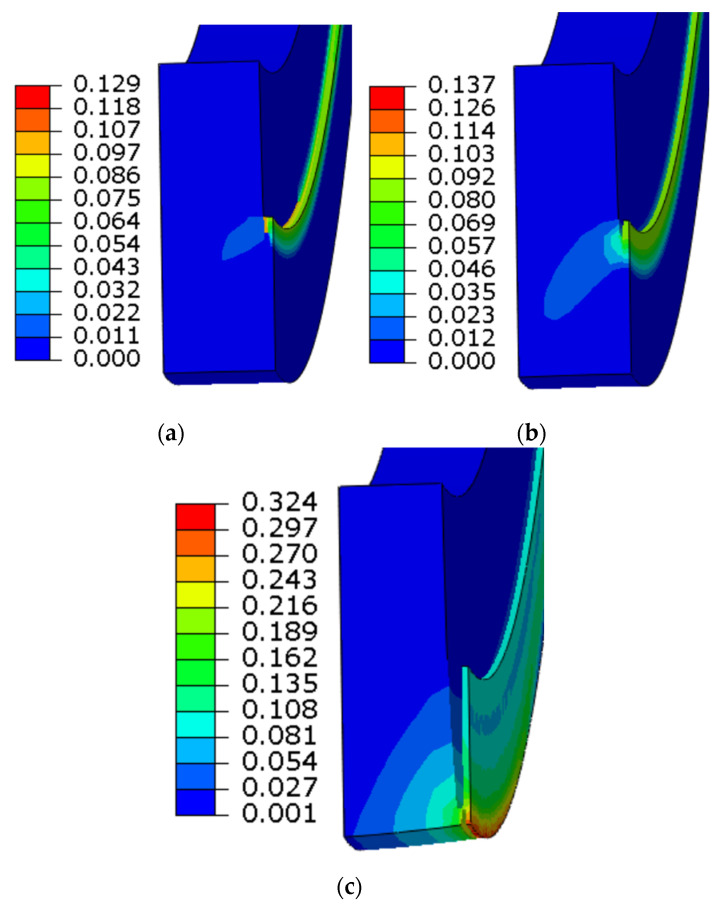
Defect failure process/equivalent plastic strain (PEEQ): (**a**) 21.5 MPa, (**b**) 21.75 MPa, (**c**) 22 MPa.

The simulation results show that the piping defect reached the critical state when the internal pressure reached 21.5 MPa, the first element in the defect failed when the internal pressure reached 21.75 MPa, and the pipe wall was penetrated at 22 MPa; the failure mode was basically consistent with the test, as shown in Figure 13. Because the pressure at which the first element of the pipe failed differed very little from the pressure that penetrated the wall, the failure pressure of the first element was taken as the failure pressure of the pipe. The simulated blasting pressure differed by only 0.23 MPa from the test result; the relative error was 1%. This shows that the finite element model based on the established fracture model was able to accurately reflect the fracture response of the actual pipeline.

## 5. Fracture Response under Pressure and Bending Moment

**Figure 14:**
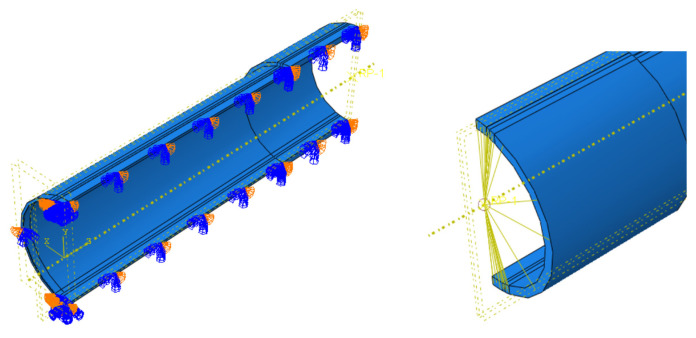
Application of boundary conditions and coupling constraints.

To obtain the fracture response of the weld defects under the combined action of internal pressure and bending moment, a maximum pipe diameter of 1422 mm and a wall thickness of 32.1 mm were taken to define the investigation object. Weld defects in the pipe girth were set as completely impermeable defects. The model was established as a quarter, and symmetric boundary conditions were imposed on the corresponding symmetry plane, as shown in Figure 14. 

**Figure 15:**
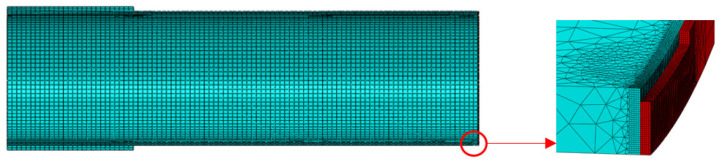
The pipe with the details of the crack.

In order to prevent the end from buckling, the end was thickened, and the pipe was set to 10,000 mm to eliminate the influence of the end effect on the stress of the pipe girth weld. Element partitions at the defect were much denser than the rest of the pipe. The overall grid size was 50 mm. The thickness direction was divided into three layers. The element size of the densified part was 1 mm, as shown in Figure 15 (the red area is the girth weld, and the blue area is the base metal). Quasi-static calculations were performed using the ABAQUS explicit dynamic, reduced integral control, and the C3D8R element, characterizing the pipeline fracture by removing the failure element.

### 5.1. Parametric Studies

In order to explore the influence of the size of the girth weld defect on the fracture of the pipeline under the coupling action, a numerical calculation of the fracture of the pipeline with a girth weld defect subjected to internal pressure and bending moment was carried out for different girth weld lengths and defect depths. The set defect depths were 6.42 mm (20% pipe wall thickness), 12.84 mm (40% pipe wall thickness), 19.26 mm (60% pipe wall thickness), and 25.68 mm (80% pipe wall thickness). The circumferential lengths of the set defects were 5%, 10%, 15%, and 20% of the circumference of the pipeline, respectively. The weld width was 10 mm. The shape of the defect corresponded to that shown in Figure 9 and was located in the middle of the bottom of the pipe. The minimum number of elements for all calculated cases was 59,298, and the maximum number of elements was 238,560.

**Figure 16:**
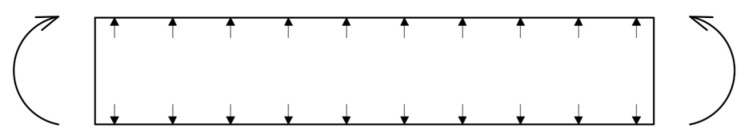
Diagram of the pipe force.

There was no rotational degree of freedom in the solid element, and the bending moment could not be directly applied to the pipe end. Therefore, a reference point was defined at the pipe end, and the degree of freedom of the pipe end was coupled with the reference point, as shown in Figure 14. The internal pressure applied to the inner wall of the pipe was 12 MPa (actual operating pressure of the pipeline). The bending moment was applied on the basis of internal pressure, and on the coupling reference point in the form of a corner until the pipe fractured. The force on the pipeline is shown in Figure 16.

### 5.2. Results

**Figure 17:**
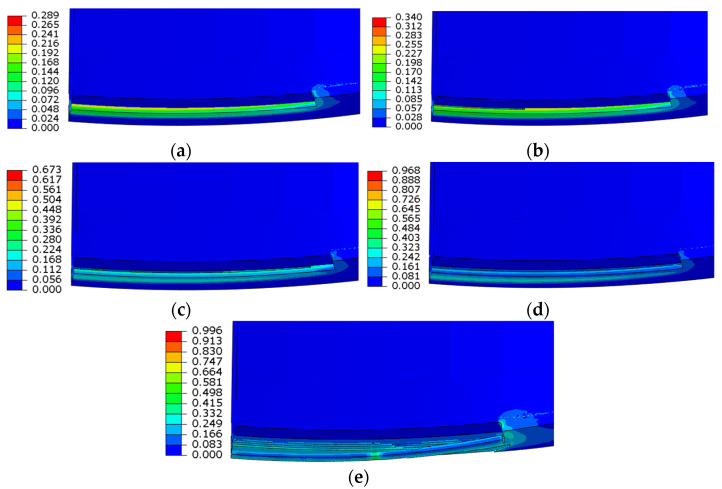
Fracture process of girth weld defects/equivalent plastic strain (PEEQ): (**a**) 16,437 kN.m, (**b**) 16,730 kN.m, (**c**) 17,003 kN.m, (**d**) 17,260 kN.m, (**e**) 17,524 kN.m.

Figure 17 shows the fracture process of the girth defect (D = 12.84 L = 10%). The fracture occurred almost instantaneously, so the failure of the first element was also used as the failure state of the pipeline. 

**Figure 18:**
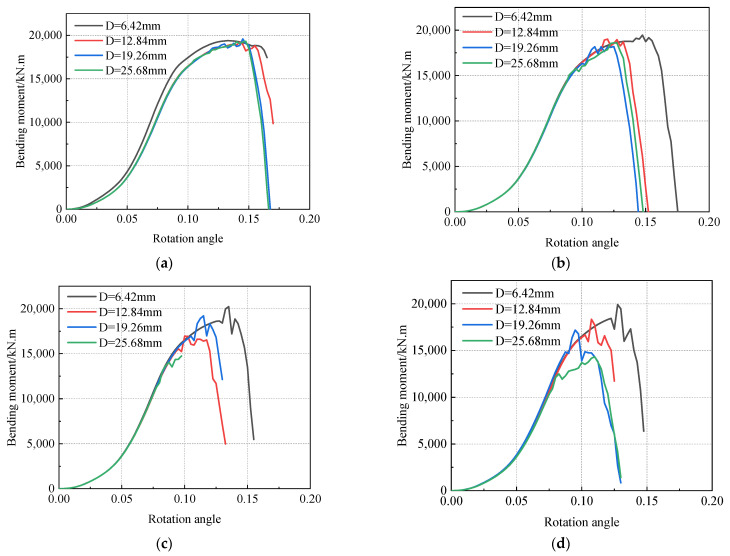
Bending moment curve under various operating conditions: (**a**) L = 5%, (**b**) L = 10%, (**c**) L = 15%, (**d**) L = 20%.

It can be seen in Figure 18 that in the application of the corner load the bending moment increased nonlinearly with the increase in the corner. The growth trend of the bending moment before the defect fracture was basically the same for each defect, and the difference was mainly shown in the fracture sequence. With the increase in defect size, the fracture point also advanced. When the circumferential length of the defect was less than 10%, the curve was relatively stable and smooth, while, when the circumferential length of the defect was 10–20%, the curve shook after fracture. It was judged that the kinetic energy after fracture was large, and the subsequent model was no longer a quasi-static process. Stress concentration occurs due to the presence of weld defects, accelerating the failure of pipelines [46,47].

**Figure 19:**
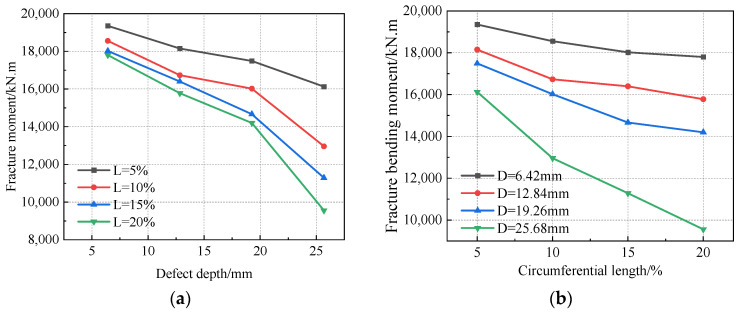
Fracture bending moments at different defect sizes: (**a**) curve of fracture bending moment with different defect lengths varying with defect depth, and (**b**) curve of fracture bending moment with different defect depths varying with defect length.

Table 2 summarizes the fracture moments for each defect. The change rule of the fracture bending moment with different defect size parameters is shown in Figure 19. The fracture bending moment was below 20,000 kN.m, and the maximum fracture bending moment of 19,348. 6 kN.m occurred when the defect size was the smallest. The minimum bending moment was 9555. 65 kN.m when the defect size was the greatest. This was only half of the maximum fracture moment. It can be seen that defect size is an important parameter affecting the fracture of pipeline girth welds. 

It can be seen in Figure 19 that the fracture bending moment decreased with increasing defect depth and circumferential length. The distance between the curves in Figure 19b is larger than that in Figure 19a, indicating that the influence of defect depth on the fracture bending moment was greater than that of the circumferential length of the defect. Among these results, the average fracture bending moment decreased by 578 kN.m when the circumferential length of the defect increased by one level, and the average fracture bending moment decreased by 1980 kN.m when the depth of the defect increased by one level. After the depth of the defect reached 25.68 mm, the change rule of the fracture moment with the circumferential length of the defect was different from that of other defect depths; that is, it was easier to fracture when the defect length reached 25.68 mm.

**Figure 20:**
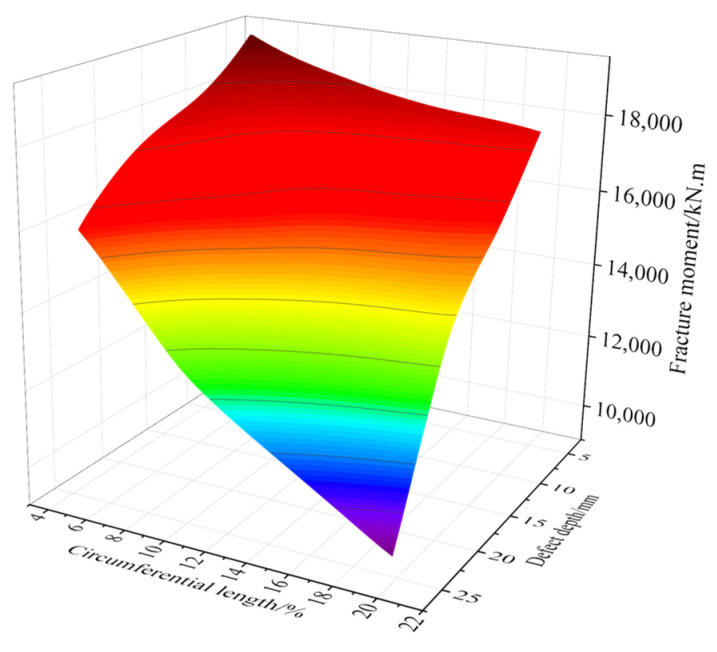
Fracture surface.

Figure 20 shows the pipeline fracture surface. When the depth of the defect was greater than 15 mm and the circumferential length was greater than 15%, the fracture bending moment showed a very small trend, and this inflection point should be considered in practical applications. However, when the defect size parameter was less than this value, the fracture bending moment presented a steady downward trend.

The fracture surface was fitted by cubic spline interpolation. The fracture prediction formula is proposed as follows:

(8) $$M=p_{00}+p_{10}D+p_{01}L+p_{20}D^{2}+p_{11}DL+p_{02}L^{2}+p_{30}D^{3}+p_{21}D^{2}L+p_{12}DL^{2}+p_{03}L^{3}$$

where *M* is the fracture moment, *D* is the weld defect depth, and *L* is the weld defect length as a percentage of pipeline circumferential length. The determined parameters are shown in Table 3.

## 6. Conclusions

It is practical and accurate to establish girth weld defects using the finite element method for evaluation, and it also solves the problems that arise when using the analytical method. The main conclusions are as follows:

(1) It was proven that the uncoupled fracture model (LSMCS) is a feasible tool for obtaining the fracture response of oil and gas pipelines.
(2) The influence of the girth weld defect size on pipeline fracture can be better obtained through parametric finite element simulation analysis. It was noted that the depth of the defect had a greater impact on the rupture of the pipeline than the circumferential length of the defect. When the defect reached 25.68 mm, the pipeline was more vulnerable to fracture. Regarding the 3D fracture surface of defect depth, defect circumferential length, and fracture bending moment, there was a sudden change point at which the pipeline became more vulnerable to fracture.
(3) This study applied the model to the fracture response under internal pressure and bending moments. This model can also be used to study the fracture responses of pipeline girth welds under other loads. The fracture responses of pipelines under more complex conditions can be considered in future work.

## Figures and Tables

**Table 1:** Mechanical parameters of X80 pipeline steel.

Young’s Modulus (MPa)	Poisson’s Ratio	Yield Strength (Rp0.2) (MPa)	Tensile Strength (MPa)
206,000	0.3	638	739

**Table 2:** Fracture bending moments at different defect sizes (kN.m).

	D (mm)	5	10	15	20
L (%)					
6.42		19,348.6	18,553.5	18,019.5	17,798.6
12.84		18,149.9	16,730.2	16,396.1	15,778.0
19.26		17,488.1	16,020.5	14,664.5	14,197.9
25.68		16,124.2	12,958.2	11,286.2	9555.65

**Table 3:** Fitting determined pending parameters (kN.m).

*P*_00_	*P*_10_	*P*_01_	*P*_20_	*P*_11_	*P*_02_	*P*_30_	*P*_21_	*P*_12_	*P*_03_
26,250	−1224	−486.3	78.82	4	26.45	−1.565	−0.972	0.433	−0.646

## Data Availability

The data used in the study is available with the authors and can be shared upon reasonable request.
